# Dynamic Changes in Sensory Quality and Chemical Components of Bingdao Ancient Tree Tea During Multiple Brewing

**DOI:** 10.3390/foods14142510

**Published:** 2025-07-17

**Authors:** Chunju Peng, Yuxin Zhao, Sifeng Zhang, Yan Tang, Li Jiang, Shujing Liu, Benying Liu, Yuhua Wang, Xinghui Li, Guanghui Zeng

**Affiliations:** 1 Wenzhou Key Laboratory of Early Sprouting Tea Breeding, Wenzhou Academy of Agricultural Sciences, Wenzhou 325006, China; chunjupeng@163.com (C.P.); tangyan@njfu.edu.cn (Y.T.); 2 Cangnan Industrial Research Institute of Modern Agriculture, Wenzhou 325800, China; wangyuhua@njau.edu.cn; 3 Tea Research Institute, Nanjing Agricultural University, Nanjing 210095, China; 2021204037@stu.njau.edu.cn (Y.Z.); 2016104090@njau.edu.cn (S.Z.); liushujing@njau.edu.cn (S.L.); 4 Jinhua Academy of Agricultural Sciences, Jinhua 321017, China; zhuwei2007100@163.com; 5 Yunnan Provincial Key Laboratory of Tea Sciences, Yunnan Academy of Agricultural Sciences, Kunming 650205, China; liusuntao@126.com

**Keywords:** Bingdao ancient tree tea, brewing times, sensory quality, chemical quality, infusion color

## Abstract

Bingdao ancient tree tea (BATT), a type of raw Pu-erh tea, is renowned for its brewing durability, characterized by a unique aroma and flavor. To explore the dynamic changes in infusion quality and the impact of multiple steeping process, BATT was brewed 14 times, and its sensory attributes, infusion color, and chemical composition were assessed across different brewing intervals. The color of the tea infusion remained relatively stable throughout the brewing process. Sensory evaluation indicated that BATT exhibited optimal sensory quality between the third and seventh infusions. While the leaching of polyphenols showed minimal variation across brews, the concentrations of ester-catechins, non-ester catechins, free amino acids, and caffeine after the seventh brewing decreased by 28.82%, 21.83%, 28.86%, and 40.37%, respectively. Our results indicated that higher concentrations of flavor compounds in the BATT infusion appeared between the fourth and seventh brews. This study provides a theoretical basis for understanding the brewing characteristics of BATT.

## 1. Introduction

According to manufacturing process, tea is classified into six categories: unfermented green tea, slightly fermented white and yellow tea, semi-fermented oolong tea, fully fermented black tea, and post-fermented dark tea [1]. Unfermented raw Pu-erh belongs to sun-dried green tea. Studies have confirmed that bioactive compounds in raw Pu-erh tea have health benefits, including hypoglycemic effects, lipid level regulation, antioxidative activity, and promotion of beneficial gut microbiota proliferation [2,3,4]. Bingdao ancient tree tea (BATT) is a type of raw Pu-erh tea native to Bing Dao village, located in Mengku Township, Shuangjiang County, Lincang Region of Yunnan Province, China. It is known as the ‘Queen’ of raw Pu-erh tea in Yunnan and is the origin of the Mengku dayezhong tea cultivar. BATT is picked from the fresh tea leaves of large-leaf tea plant species (*Camellia sinensis* (Linn.) var. *assamica* (Masters) Kitamura) through traditional processing steps, including spreading out, de-enzyming (fixation), rolling and sun-drying, and further autoclaving and compressing process [5]. Generally, the raw Pu-erh tea produced from the fresh tea leaves of Yunnan arbor-type tea trees with a growth age over 100 years is regarded as an ancient tree tea. Previous studies have revealed the chemical differences between ancient tea and terrace tea [6] as well as higher levels of quality components in large-leaf green tea than those in ordinary small-leaf species [7]. In addition to the tea quality difference caused by the raw materials, storage year, and production area, the brewing conditions, such as water temperature, water quality, and brewing times, are other important factors that affect the quality of tea infusion [8,9,10]. Therefore, the brewing times are particularly critical [11]. However, at present, limited research has focused on the quality characteristics of BATT and other ancient tree teas and the dynamic changes in tea infusions during the brewing process remain unclear.

Sensory evaluation is the most conventional method for judging the taste and aroma of tea infusion [12]. Notably, taste stability is a crucial criterion for determining whether the tea infusion is suitable for consumption over multiple brewing. Therefore, it is essential to analyze the sensory quality of tea infusion throughout the brewing process. In addition, tea infusion color is an integral component of sensory evaluation, which can help us intuitively judge the change in tea infusion quality. For instance, prolonged storage has been shown to alter the color and quality of raw Pu-erh tea infusions [13].

The taste of tea and its health benefits are determined by the functional components of tea infusions [14]. The functional components that form the flavor of tea infusion are caffeine, polyphenols, amino acids, sugars, and various aromatic substances. Polyphenols, dominated by catechins, are the main nutritional components in green tea and have been identified as great contributions to the flavor of tea infusions [15]. Caffeine also enhances tea flavor and induces bitter taste of tea infusion without activating bitter taste receptors [16,17]. Amino acids and soluble sugars (e.g., sucrose, glucose, and fructose) serve as primary sources of sweetness in tea infusions, playing an important role in neutralizing their bitterness [18]. Furthermore, free amino acids, especially L-theanine and L-glutamic acid, contributed approximately 70% of the umami taste intensity in tea [19].

The optimal brewing times varied among tea types. For instance, green tea is typically recommended to be brewed up to four times [20]. However, the quality of tea infusion is determined by both chemical composition and sensory quality, with this combination further influenced by tea cultivars, raw materials, and processing methods [10]. Thus, scientific brewing protocols are required to maximize the quality of tea infusions for specific tea types. The aim of this study was to investigate the dynamic changes in BATT quality during the brewing process and elucidate its characteristics of brewing durability, thereby providing the optimal brewing protocols of BATT.

## 2. Materials and Methods

### 2.1. Sample Preparation and Collection

The BATT samples were collected from the ancient tea garden in Iceland Village, Yunnan Province, China, and provided by the Shuangjiang Mengku Iceland Huali Ancient Tree Tea Company. All of the tea samples were produced by one bud and two leaves from an ancient tea tree in the spring. The tea samples used in this study were stored at 4 °C for sensory evaluation and chemical determinations.

### 2.2. Chemicals and Reagents

Gallic acid (CAT#RQ0200, 98.5%) and L-Theamine (CAT#RA1123, 99.0%) standards were purchased from Ruiyong Biotechnology Co., Ltd. (Shanghai, China). Caffeine (CAT#S59724, 99%), gallocatechin (GC, CAT#B30661, 98%), epicatechin gallate (ECG, CAT#B20103, 98%), epicatechin (EC, CAT#20102, 98%), gallate (EGCG, CAT#B20106, 98%), epigallocatechin (EGC, CAT#B20105, 98%), catechin gallate (CG, CAT#B20350, 98%), gallocatechin gallate (GCG, CAT#20850, 98%), and catechin (C, CAT#A10025, 99.8%) standards, as well as Folin–Phenol were purchased from Yuanye Biotechnology Co., Ltd. (Shanghai, China). Acetonitrile, methanol, and disodium ethylenediamine tetraacetate (EDTA) were purchased from Jewinda Biotechnology Co., Ltd. (Nanjing, China).

### 2.3. Preparation of Brewed Tea

The tea infusions were prepared according to the previous method [11]. Briefly, 5.0 g of BATT samples were weighed and placed into a 110 mL tea bowl, and 110 mL of purified water (100 °C) was added. The tea samples were steeped 14 times for sensory evaluation and chemical composition detection of each infusion. The steeping durations at different brews were as follows: 20, 30, 45, 60, 80, 100, 120, 150, 180, 220, 260, 300, 340, and 380 s. The tea infusion was obtained and immediately cooled to room temperature in an ice water bath [21]. This process was repeated in triplicate.

### 2.4. Sensory Evaluation of Tea Infusion

The Chinese National Standard methods were used to assess every batch sample (GB/T 23776-2018) [22]. For sensory evaluation, nine trained panelists (two males and seven females) with tea evaluation expertise were recruited. The panel comprised six professional experts of tea evaluation and three professionally trained consumers. BATT was brewed 14 times, orderly served in evaluation cups, and presented to each of the panelists for sensory evaluation conducted according to the Chinese National Standard. Scores were assigned for color, aroma, and taste on a percentile system from 0 (extremely dislike) to 100 (extremely like). The participants rinsed their mouths with purified water before the next evaluation. Each evaluation factor was weighted as follows: taste (40%), aroma (40%), and infusion color (20%). The scoring criteria are shown in Table 1. The evaluation for each tea sample was re-verified in triplicate, with the average calculated as a final score.

### 2.5. Detection of Catechins and Caffeine by HPLC

According to the national method established by the China National Institute of Standardization (CNIS) [23], catechins and caffeine were detected by Prominence LC-20A high-performance liquid chromatography (HPLC) (Shimadzu Co., Ltd., Kyoto, Japan) consisting of infinity binary pump, integrated vacuum degasser, autosampler, thermos-stated column compartment, and diode array detector (DAD). The detection wavelength was set to a wavelength of 278 nm. An Agilent C18 chromatogram column (250 × 4.6 mm, 5 μm; Agilent Technologies, Santa Clara, CA, USA) with mobile phase A (90 mL acetonitrile, 20 mL acetic acid and 2 mL EDTA) and mobile phase B (800 mL acetonitrile, 20 mL acetic acid and 2 mL EDTA) as mobile phases were employed for HPLC separation with an injection volume of 10 µL. Before injection, the tea infusions were filtered through a 0.22 µm filter. The tea chromatographic conditions were set according to the previous methods [11]. The gradient eluted program was as follows: mobile phase flow rate of 1 mL/min, parameter setting of 0.01 min for 100% A; 10 min 100% A; 10.01 min 100% A, 25 min 68% A, 32% B; 25.01 min 68% A, 32% B; 35 min 68% A, 32% B; 35.01 min 100% A; and 45 min 100% A. A blank run was conducted upon the stabilization of flow rate and column temperature. A 10 µL mixed standard series working solution was injected into the HPLC to obtain calibration curves. After standardization, a 10 µL sample infusion was used for detection. The validation parameters (linearity, curve equation, correlation coefficient, and limit of detection) for the HPLC determination of catechins monomers are shown in Table S2.

### 2.6. Color Measurement of Tea Infusions

The color of tea infusions was detected by using 3nh colorimeter in transmittance mode (Sanenshi Technology, Shenzhen, China). The CIE-L*a*b* method was employed to distinguish color difference among various tea infusions [24]. The color changes across brewing times were analyzed according to the previous methods [25]. Briefly, the L*C*h° values were calculated from L*a*b* values. L* represents lightness, where a value of 100 corresponds to absolute white and 0 to absolute black. Chroma (C*) measures saturation of infusion. Hue angle (h°) is expressed on a 360° color wheel, with 0°, 90, 180°, and 270° indicating +a* (red), +b* (yellow), −a* (green), and −b* (blue), respectively. Color changes across brewing times were analyzed by measuring the lightness (L), chroma (C), and hue angle (h) of each infusion.

### 2.7. Detection of Total Free Amino Acids and Total Polyphenols

According to the GB/T8314-2013 standard, the total content of free amino acids was detected by the ninhydrin colorimetric method [26]. Briefly, 1 mL of tea infusion was transferred to a test tube, followed by a sequential addition of 0.5 mL phosphate-buffer solution (pH 8.0) and 1.0 mL of 2% (*w/v*) ninhydrin solution. The mixture was then heated in a boiling water bath for 15 min. After cooling, absorbance was measured with UV-5100 ultraviolet-visible (UV-Vis) spectrophotometer (Yuanxi Instrument, Shanghai, China) at 570 nm using the L-theanine standard curve.

The total polyphenols were detected by the Folin–Ciocalteu method [27]. Briefly, 1 mL of tea infusion was transferred to a test tube, after which it was mixed with 5 mL of Folin–Ciocalteu. After 8 min of reaction, 4.0 mL of 7.5% (*w/v*) Na_2_CO_3_ solution was added. The mixture was incubated at room temperature for 60 min in the dark. Absorbance was measured with a UV-Vis spectrophotometer (Yuan Xi Instrument, Shanghai, China) at 765 nm using the gallic acid standard curve.

### 2.8. Statistical Analysis

The data averages and standard deviations were calculated using Excel 2016. One-way analysis of variance (ANOVA) with Duncan test was performed using IBM SPSS Statistics 20 to obtain significant variations (*p* < 0.05). CIE L* a* b* was used for color difference analysis. The principal component analysis (PCA) was used to analyze the changes in chemical components during the brewing process. All experiments were repeated three times and the results were presented as means ± standard deviation (SD).

## 3. Results and Discussion

### 3.1. Dynamic Changes in Sensory Quality During Brewing

**Figure 1:**
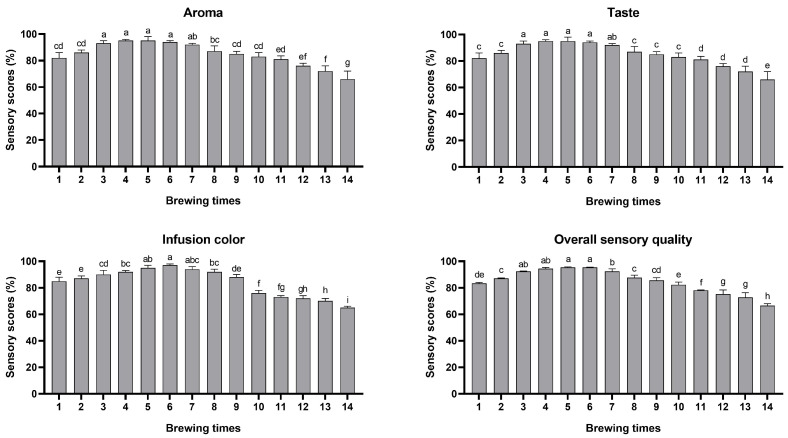
The sensory evaluation of BATT infusions throughout the brewing process. The data are presented as means ± SD. Different letters indicate significant differences at *p* < 0.05.

Brewing conditions significantly affect the concentration of functional components in tea, particularly green tea, infusion [28]. In conventional brewing practices, the concentration of tea infusion gradually decreases with increasing brewing times, resulting in diminished flavor intensity. Consequently, steeping durations of tea fusion also need to be continuously extended to maintain the intensity of the flavor. Our experiment simulated this progressive extension of steeping durations to align the daily brewing practices. BATT has unique qualities, including a distinct icing sugar and honey aroma, green and yellow clear infusion color, and strong and thick taste [29]. In this study, the aroma, taste, and infusion of BATT was evaluated according to the standard indicated in Table 1. The results of the sensory evaluation showed that the aroma, taste, and infusion color of BATT altered significantly throughout the brewing process. The scoring results of each brewing time in the aroma, taste, infusion color, and overall sensory quality are shown in Figure 1 and Table S1. We found that the aroma factor scores of BATT in the top eleven brews ranged from 80 to 100, exhibiting long-lasting aroma characteristic. The taste scores were significantly higher (exceeding 90) during between the third and the seventh brews compared with other brews. The infusion color of BATT at the sixth brew achieved the highest score, exhibiting a bright yellow color. The scores of different sensory factors followed similar trends during the whole brewing process, and the third to seventh infusions showed superior taste, aroma, and color. These results suggested that the BATT must be brewed up to seven times (for approximately 455 s) to achieve optimal flavor in most cases.

Previous studies have reported that Korean green tea could be continuously brewed five times, with the first and second infusions exhibiting the best aroma and sensory quality [20]. However, the comprehensive sensory scores showed that the sensory score of the top eleven brews was greater than 80. The sensory quality of BATT increased significantly as brewing times increased until the sixth brew, after which it gradually decreased. These results showed the stable sensory quality of BATT throughout the brewing process, which demonstrated its characteristics of high brewing durability.

### 3.2. Changes in the Color of Tea Infusions During Brewing

**Figure 2:**
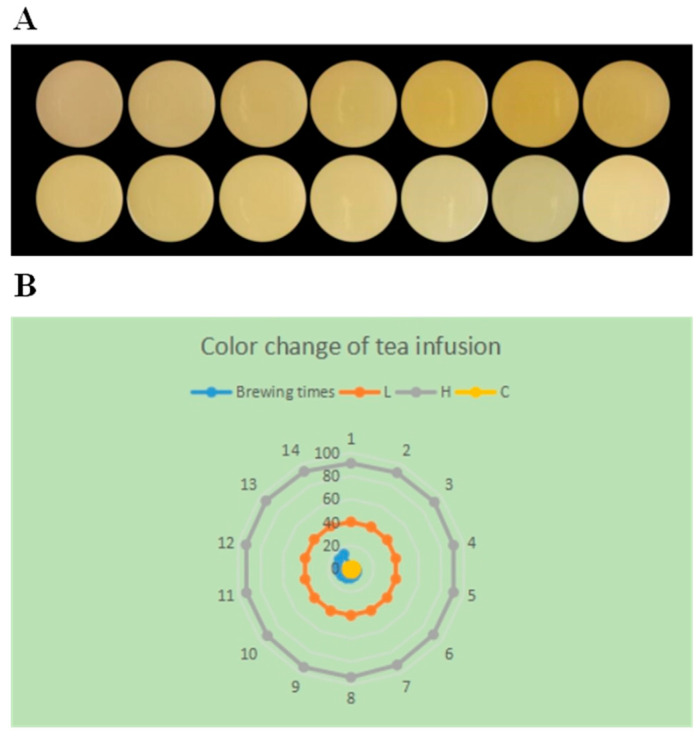
The color change (**A**), and L, H, and C values (**B**) of tea infusion during the brewing process. L, H, and C represent the lightness, saturation, and hue, respectively.

The color variation in BATT infusion across 14 brewing times is shown in Figure 2A. During the entire brewing process, the color of the tea infusion gradually changed from green–yellow to yellow, and then back to light green–yellow. The color of tea infusion gradually deepened until the seventh brews, after which it began to lighten.

To clarify the dynamic changes in infusion color during the brewing process, we used the CIE L* a* b* method to test the lightness (L), saturation (C), and hue angle (H) values of tea infusion. The color difference values of L, H, C, ‘a’, and ‘b’ are commonly used as indicators to analyze color changes in tea infusions. Among them, L represents the lightness of the tea infusion. C represents the saturation of the color of the tea infusion, indicating the color concentration and chroma intensity of tea infusion. H represents the hue angle of tea infusion. The hue angle is generated by the change in the wavelength of light waves, which can distinguish the color difference in the same color system [24]. The ‘a’ value represents the red–green degree, where positive values indicate red and the negative values indicate green. The ‘b’ value represents the yellow–blue degree, where the positive values indicate yellow and the negative values indicate blue [25].

As shown in Table 2, the ‘a’ value of tea infusion was negative throughout the brewing process, and its absolute value gradually increased with the increase in brewing times. Our results demonstrated that the color of tea infusion was green-based during the brewing process. However, no significant difference was found in the ‘a’ value across 14 brewing times, indicating that brewing time had little influence on the red–green balance of BATT. This result is similar to that found by Lin et al. [30]. The ‘b’ value of tea infusion was positive throughout the brewing process. Notably, the yellowness value was significantly higher at the fourth to seventh brews than that at other brews. Our results suggested that the ‘a’ and ‘b’ values of color difference were consistent with the color change in Figure 2A.

The L, H, and C values of the tea infusion obtained from 14 brews are shown in Figure 2B. No significant difference was found in lightness, saturation, or hue angle of tea infusion during the brewing process. The lower lightness and saturation of tea infusion indicated that the color of BATT infusion was relatively simple. Figure 2B showed an irregularly circular pattern, indicating that there was a difference in color of the tea infusion during the brewing process, but the overall change was small. In addition, hue angle, a primary characteristic of infusion color, did not change significantly during the brewing process. These results suggested that the color of BATT infusion did not change significantly during the brewing process, further demonstrating the stable quality of BATT infusion.

### 3.3. Leaching Dynamics of Chemical Composition in Tea Infusion During Brewing

**Figure 3:**
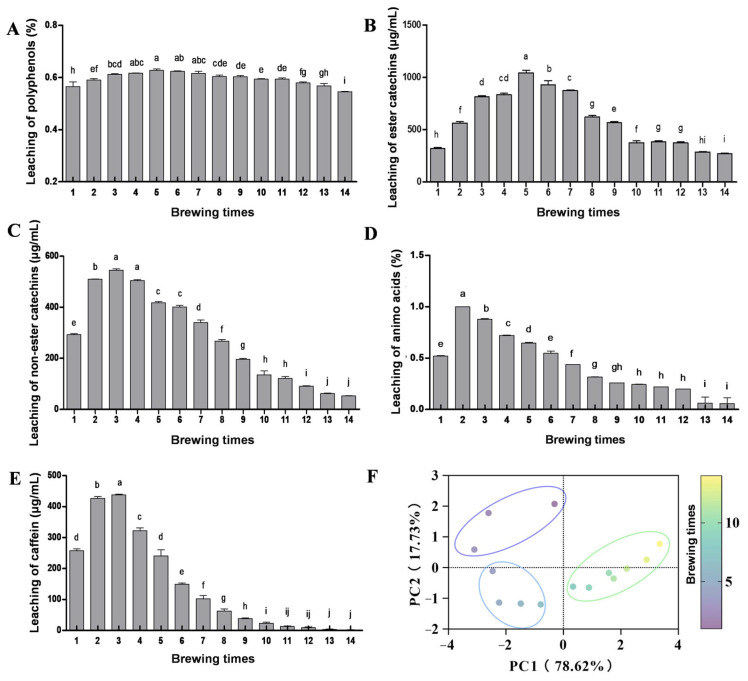
Leaching of polyphenols (**A**), ester (**B**) and non-ester (**C**) catechins, amino acids (**D**), and caffeine contents (**E**) of Bingdao ancient tree tea fusions during the brewing process. (**F**) Principal component analysis of Bingdao ancient tree tea samples. Changes in icon color represent change in brewing times. The separation of different samples indicates significant differences between samples and brewing times. The data are presented as means ± SD. The different letters indicate significant differences at *p* < 0.05.

To understand the leaching dynamics of quality components during the brewing process, the leaching of total polyphenols, amino acids, caffeine, and catechin monomers in each BATT infusion was detected (Figure 3). During the whole brewing process, the leaching of polyphenols in each tea infusion never fell below 0.5%. The leaching of the total polyphenols in each tea infusion gradually increased through the first five brews, and then gradually decreased from the sixth to fourteenth brews. However, the leaching trends during the whole brewing process were different between ester catechins (i.e., ECG, EGCG, CG, and GCG) and non-ester catechins (i.e., GC, EC, C, and EGC). The leaching of ester catechins increased to the highest level at the fifth brews, while the leaching of non-ester catechins increased to the highest level at the third brews, indicating a higher leaching rate for non-ester catechins compared to ester catechins. In addition, the leaching amount of ester catechins during the brewing process was significantly higher than that of non-ester catechins, which might be related to the higher contents of ester catechins in tea leaves. Ester catechins, particularly EGCG, have a greater contribution to the astringency of tea infusion, whereas non-ester catechins have a weaker astringent taste [31,32]. These results explained why the BATT infusion had strong astringency after multiple brewing.

For other quality components, the leaching of total amino acids in tea infusion peaked at the second brew, after which it decreased significantly. The leaching of caffeine varied significantly across brewing times, decreasing rapidly after the third brew and falling below 10 mg/mL by the fourteenth brew, possibly because caffeine is easier to be soaked out and dissolved into the tea infusion quickly [33]. Amino acids primarily contribute to umami, while caffeine contributes to bitterness. Notably, leaching analysis revealed that the higher leaching of each chemical component appeared within the first seven brews. After the seventh brew, the contents of amino acids and caffeine decreased significantly, while the content of total polyphenols exhibited smaller variations, suggesting that the bitter and astringent characteristics of the tea infusion had become more pronounced, thus diminishing the sensory quality.

The resistance of tea infusion to brewing was reported to have a significant positive correlation with the chemical composition [11]. As brewing times and steeping duration increased, the concentrations of chemical components in tea infusion generally rose initially and then declined. After the initial wetting during the first brew, non-ester catechins, amino acids, and caffeine in BATT infusions underwent rapid leaching at the subsequent second and third brews, with a steeping duration of 30 and 45 s, respectively, indicating that short steeping was sufficient to achieve optimal sensory quality in the early brewing stages. As steeping duration increased from 60 s (fourth brew) to 120 s (seventh brew), the leaching rates of non-ester catechins and amino acids declined gradually, while those of ester catechins exhibited a progressive increase. The dynamic changes in these components maintained the flavor balance of BATT infusion. However, after multiple brewing, even with the steeping duration being extended to 380 s at the 14th brews, it failed to enhance the leaching rates of chemical components. To distinguish the quality differences among different brews, PCA was conducted on different chemical components. Although the leaching patterns of chemical components during the brewing process were different, our results demonstrated that the tea infusion samples could be grouped into three distinct clusters. Notably, tea infusions from the fourth to the seventh brews formed a single cluster, aligning with the optimal sensory brewing range. This result further confirmed that tea infusions from the fourth to seventh brews exhibited higher nutritional value and better flavor. Consistent trends in chemical and sensory quality provided evidence for the drinking value of BATT. It should be noted that the BATT sample used in this study was produced within one year. These conclusions may not represent the brewing durability of BATT from different storage years. Therefore, further investigation into BATT quality across storage years is required to comprehensively understand the brewing characteristics of BATT.

## 4. Conclusions

In this study, we comprehensively examined the effects of multiple steeping processes on the sensory quality, the change in infusion color, and chemical components of BATT. Our results showed that BATT achieved optimal sensory quality between the third and seventh brews. Color difference analysis revealed no significant changes in lightness, saturation, or hue angle throughout the brewing process. Although the levels of polyphenols in the whole brewing process remained relatively stable, the concentrations of catechins, caffeine, and free amino acids declined sharply after the seventh infusion. These results confirm the strong infusion durability of BATT and highlight that higher concentrations of flavor compounds and sensory quality of BATT occur between the fourth and seventh brews. This study provides a theoretical basis for understanding the brewing durability of BATT during repeated infusions.

## Figures and Tables

**Table 1:** The sensory evaluation standard of Bingdao ancient tree tea.

Sensory Factors	Description of Sensory Characteristics	Corresponding Scores (%)
Aroma	Strong fruity flowery, honey fragrance, elegant fragrance, long-lasting	90–100
	Honey fragrance, long-lasting	80–90
	Pure and normal	70–80
	Short fragrant	60–70
	Unobvious short aroma	50–60
	No aroma	40–50
Taste	Strong and thick, smooth, obvious sweet after taste	90–100
	Strong and approach thick, sweet after taste	80–90
	Mellow, have sweet after taste	70–80
	Mellow and thin, have sweet after taste	60–70
	Plain and thin	50–60
	Dull like water	40–50
Infusion color	Greenish yellow, bright	90–100
	Yellowish green, bright	80–90
	Light yellow, bright	70–80
	Yellowish	60–70
	Yellowish, light	50–60
	Light	40–50

**Table 2:** The effects of brewing times on the infusion color of Bingdao ancient tree tea.

Brewing Times	Red–Green Degree	Yellow–Blue Degree
	a	b
1	−0.13 ± 0.44 ^a^	1.62 ± 0.31 ^h^
2	−0.15 ± 0.20 ^a^	2.91 ± 0.46 ^efg^
3	− 0.12 ± 0.16 ^a^	3.39 ± 0.18 ^cde^
4	−0.04 ± 0.22 ^a^	3.82 ± 0.42 ^abc^
5	−0.06 ± 0.14 ^a^	3.91 ± 0.0.22 ^abc^
6	−0.07 ± 0.20 ^a^	4.24 ± 0.01 ^a^
7	−0.13 ± 0.18 ^a^	4.19 ± 0.65 ^ab^
8	−0.20 ± 0.21 ^a^	3.69 ± 0.65 ^abcd^
9	−0.25 ± 0.16 ^a^	3.76 ± 0.43 ^abcd^
10	−0.13 ± 0.15 ^a^	3.46 ± 0.50 ^bcde^
11	−0.13 ± 0.20 ^a^	3.32 ± 0.39 ^cdef^
12	−0.14 ± 0.15 ^a^	3.04 ± 0.36 ^defg^
13	−0.17 ± 0.15 ^a^	2.64 ± 0.20 ^fg^
14	−0.12 ± 0.16 ^a^	2.45 ± 0.27 ^g^

Different lowercase letters indicate significant differences at *p* < 0.05.

## Data Availability

The original contributions presented in this study are included in the article; further inquiries can be directed to the corresponding author.

## Supplementary Materials

The following supporting information can be downloaded at https://www.mdpi.com/article/10.3390/foods14142510/s1. Table S1: The sensory evaluation of Bingdao ancient tea infusions across 14 brewing times (%). Table S2: Validation parameters for the HPLC determination of catechins monomers.

## References

[1] Zhang Y.D., Yang X.J., Cattani C., Rao R., Wang S.H., Phillips P. (2016). Tea category identification using a novel fractional fourier entropy and jaya algorithm. Entropy.

[2] Ding Q.Z., Zheng W., Zhang B.X., Chen X.J., Zhang J., Pang X., Zhang Y., Jia D.X., Pei S.R., Dong Y.S. (2019). Comparison of hypoglycemic effects of ripened pu-erh tea and raw pu-erh tea in streptozotocin-induced diabetic rats. RSC Adv..

[3] Deng X.J., Zhang N., Wang Q.G., Huang Y.Y., Huang Y.L., Lin Y.P., Huang M.J., Zheng F.M., Xiao M.T., Ye J. (2023). Theabrownin of raw and ripened pu-erh tea varies in the alleviation of hfd-induced obesity via the regulation of gut microbiota. Eur. J. Nutr..

[4] Lee L.K., Foo K.Y. (2013). Recent advances on the beneficial use and health implications of pu-erh te. Food Res. Int..

[5] Lv H.P., Zhang Y.J., Lin Z., Liang Y.R. (2013). Processing and chemical constituents of pu-erh tea: A review. Food Res. Int..

[6] Zhang S.R., Shi Y., Jiang J.L., Luo L.Y., Zeng L. (2022). Discriminant analysis of Pu-erh tea of different raw materials based on phytochemicals using chemometrics. Foods.

[7] Cao X.X., Liu M.M., Hu Y.J., Xue Q., Yao F., Sun J., Sun L.W., Liu Y.J. (2021). Systemic characteristics of biomarkers and differential metabolites of raw and ripened Pu-erh teas by chemical methods combined with a UPLC-QQQ-MS-based metabolomic approach. LWT.

[8] Pérez-Burillo S., Giménez R., Rufián-Henares J., Pastoriza S. (2018). Effect of brewing time and temperature on antioxidant capacity and phenols of white tea: Relationship with sensory properties. Food Chem..

[9] Xu Y.Q., Zou C., Gao Y., Chen J.X., Wang F., Chen G.S., Yin J.F. (2017). Effect of the type of brewing water on the chemical composition, sensory quality and antioxidant capacity of Chinese teas. Food Chem..

[10] Liu Y.Q., Feng X.Y., Gao T., Pan Y.N., Lv H.L., Chen M., Shen Y.L., Zhu W., Yao Y.Y., He L.X. (2024). Advancement and challenges in tea brewing: The dynamic principles, influencing factors, innovative processing technologies and pollutants. Trends Food Sci. Tech..

[11] Zhang S.F., Yang Y.Q., Cheng X.F., Thangaraj K., Arkorful E., Chen X., Li X.H. (2020). Prediction of suitable brewing cuppages of dahongpao tea based on chemical composition, liquor colour and sensory quality in different brewing. Sci. Rep..

[12] Zeng L., Fu Y.Q., Liu Y.Y., Huang J.S., Chen J.X., Yin J.F., Jin S., Sun W.J., Xu Y.Q. (2023). Comparative analysis of different grades of tieguanyin oolong tea based on metabolomics and sensory evaluation. LWT.

[13] Gao L., Bian M.X., Mi R.F., Hu X.S., Wu J.H. (2016). Quality identification and evaluation of Pu-erh teas of different grade levels and various ages through sensory evaluation and instrumental analysis. Int. J. Food Sci. Tech..

[14] Chaturvedula V.S.P. (2011). and Prakash, I. The aroma, taste, color and bioactive constituents of tea. J. Med. Plants Res..

[15] Kerio L., Wachira F.N., Wanyoko J.K., Rotich M. (2013). Total polyphenols, catechin profiles and antioxidant activity of tea products from purple leaf coloured tea cultivars. Food Chem..

[16] Yu P.G., Yeo A.S., Low M.Y., Zhou W.B. (2014). Identifying key non-volatile compounds in ready-to-drink green tea and their impact on taste profile. Food Chem..

[17] Saklar S., Ertas E., Ozdemir I.S., Karadeniz B. (2015). Effects of different brewing conditions on catechin content and sensory acceptance in turkish green tea infusions. J. Food Sci. Tech..

[18] Li M.J., Ma J.P., Li Q.J., Zhu Y.Y., Xu H., Ye N.X., Wang F.Q., Jin S. (2024). Analysis of pivotal compounds in nanlushuixian tea infusion that connect its color and taste. J. Food Compos. Anal..

[19] Zhang H.H., Li Y.L., Lv Y.J., Jiang Y.L., Pan J.X., Duan Y.W., Zhu Y.J., Zhang S.K. (2017). Influence of brewing conditions on taste components in fuding white tea infusions. J. Sci. Food Agric..

[20] Lee J., Chambers D., Chambers IV E. (2013). Sensory and instrumental flavor changes in green tea brewed multiple times. Foods.

[21] Liang Y., Zhang L., Lu J. (2005). A study on chemical estimation of pu-erh tea quality. J. Sci. Food Agric..

[22] (2018). Methodology for Sensory Evaluation of Tea.

[23] (2008). Determination of Total Polyphenols and Catechins Content in Tea.

[24] Wrolstad R.E., Durst R.W., Lee J. (2005). Tracking color and pigment changes in anthocyanin products. Trends Food Sci. Tech..

[25] Sui X.N., Bary S., Zhou W.B. (2016). Changes in the color, chemical stability and antioxidant capacity of thermally treated anthocyanin aqueous solution over storage. Food Chem..

[26] Tu Y.X., Bian M., Wan Y.K., Fei T. (2018). Tea cultivar classification and biochemical parameter estimation from hyperspectral imagery obtained by UAV. PeerJ.

[27] Blainski A., Lopes G.C., De Mello J.C.P. (2013). Application and analysis of the Folin-ciocalteu method for the determination of the total phenolic content from limonium brasiliense l. Molecules.

[28] Deng S.H., Cao Q.Q., Zhu Y., Wang F., Chen J.X., Zhang H., Granato D., Liu X.H., Yin J.F., Xu Y.Q. (2023). Effects of natural spring water on the sensory attributes and physicochemical properties of tea infusions. Food Chem..

[29] Xiong Z.C., Feng W.Z., Xia D.Z., Zhang J.X., Wei Y.M., Li T.H., Huang J.L., Wang Y.J., Ning J.M. (2023). Distinguishing raw Pu-erh tea production regions through a combination of hs-spme-gc-ms and machine learning algorithms. LWT.

[30] Lin S.D., Yang J.H., Hsieh Y.J., Liu E.H., Mau J.L. (2014). Effect of different brewing methods on quality of green tea. J. Food Process Preserv..

[31] Yang G.Z., Meng Q., Shi J., Zhou M.X., Zhu Y., You Q.S., Xu P., Wu W.L., Lin Z., Lv H.P. (2023). Special tea products featuring functional components: Health benefits and processing strategies. Compr. Rev. Food Sci. Food Saf..

[32] Zhuang J.H., Dai X.L., Zhu M.Q., Zhang S.X., Dai Q.Y., Jiang X.L., Liu Y.J., Gao L.P., Xia T. (2020). Evaluation of astringent taste of green tea through mass spectrometry-based targeted metabolic profiling of polyphenols. Food Chem..

[33] Price W.E., Spiro M. (1985). Kinetics and equilibria of tea infusion: Theaflavin and caffeine concentrations and partition constants in several whole teas and sieved fractions. J. Sci. Food Agric..

