# Supplementary material for: Dynamic Changes in Sensory Quality and Chemical Components of Bingdao Ancient Tree Tea During Multiple Brewing
Source: Foods. 2025 Jul 17;14(14):2510. doi: 10.3390/foods14142510 (PMC12294966; doi:10.3390/foods14142510)
Supplement: Supplementary file 1 [file foods-14-02510-s001.zip › foods-3699734-supplementary.pdf]

Table S1. The sensory evaluation of Bingdao ancient tea infusions at 14 brewing times (%).

Brewing times	aroma	taste	Infusion color	Overall sensory quality
1	82 ± 4.00^{cd}	84 ± 1.00^{c}	85 ± 3.00^{e}	83.4 ± 0.64^{de}
2	86 ± 2.00^{cd}	88 ± 2.00b^{c}	87 ± 2.00^{e}	87 ± 0.40^{c}
3	93 ± 2.00^{a}	93 ± 1.00^{a}	90 ± 3.10^{cd}	92.4 ± 0.23^{ab}
4	95 ± 1.00^{a}	95 ± 2.00^{a}	92 ± 1.00^{bc}	94.4 ± 1.00^{ab}
5	95 ± 3.00^{a}	96 ± 1.00^{a}	95 ± 2.00^{ab}	95.4 ± 0.40^{a}
6	94 ± 1.00^{a}	96 ± 1.00^{a}	97 ± 1.00^{a}	95.4 ± 0.20^{a}
7	92 ± 1.00^{ab}	92 ± 3.10^{ab}	94 ± 2.00^{abc}	92.4 ± 2.01^{b}
8	87 ± 4.00^{bc}	86 ± 2.00^{c}	92 ± 2.00^{bc}	87.6 ± 2.00^{c}
9	85 ± 2.00^{cd}	85 ± 2.00^{c}	88 ± 2.00^{de}	85.6 ± 2.00^{cd}
10	83 ± 3.00^{cd}	85 ± 3.00^{c}	76 ± 2.00^{f}	82.4 ± 2.00^{e}
11	81 ± 2.52^{ed}	78 ± 2.00^{d}	73 ± 1.00^{fg}	78.2 ± 0.16^{f}
12	76 ± 2.00^{ef}	76 ± 5.00^{d}	72 ± 2.00^{gh}	75.2 ± 3.20^{g}
13	72 ± 4.00^{f}	75 ± 4.00^{d}	70 ± 2.00^{h}	72.8 ± 3.60^{g}
14	66 ± 6.00^{g}	68 ± 3.00^{e}	65 ± 1.00^{i}	66.6 ± 1.40^{h}

Notes: Data are presented as means ± SD. Different letters indicate significant differences at $P < 0.05$.

Table S2. Validation parameters for the HPLC determination of catechins monomers.

Compounds	Linearity (μg/mL)	Curve equation	Correlation Coefficient (R^2)	Limit of detection （μg/mL）
GA	0.05-50	y = 28288x - 122492	0.9997	0.012
GC	0.01-10	y = 2218.2x - 46108	0.9985	0.098
CAF	0.01-10	y = 24487x + 309337	0.9965	0.032
EGC	0.2-200	y = 2184.1x - 31830	0.9991	0.065
C	0.05-50	y = 6847.1x - 174193	0.9991	0.073
EGCG	0.35-350	y = 10853x - 706350	0.9901	0.006
EC	0.5-50	y = 7694.1x - 160514	0.9988	0.046
GCG	0.05-50	y = 9619.9x - 795167	0.9945	0.039
ECG	0.05-50	y = 12467x - 786792	0.9914	0.014
CG	0.05-50	y = 12211x - 998277	0.9966	0.022
